# Clinical outcomes and risk factors for immune recovery and all‐cause mortality in Latin Americans living with HIV with virological success: a retrospective cohort study

**DOI:** 10.1002/jia2.26214

**Published:** 2024-03-17

**Authors:** Gabriel Castillo‐Rozas, Shengxin Tu, Paula Mendes Luz, Fernando Mejia, Juan Sierra‐Madero, Vanessa Rouzier, Bryan E. Shepherd, Claudia P. Cortes

**Affiliations:** ^1^ Laboratory of Molecular and Cellular Virology Institute of Biomedical Sciences Faculty of Medicine University of Chile Santiago Chile; ^2^ HIV/AIDS Workgroup, Faculty of Medicine University of Chile Santiago Chile; ^3^ Department of Biostatistics Vanderbilt University Medical Center Nashville Tennessee USA; ^4^ Evandro Chagas National Institute of Infectious Diseases Oswaldo Cruz Foundation Rio de Janeiro Brazil; ^5^ Instituto de Medicina Tropical Alexander von Humboldt Universidad Peruana Cayetano Heredia Lima Perú; ^6^ Instituto Nacional de Ciencias Médicas y Nutrición Salvador Zubirán Ciudad de México México; ^7^ Groupe Haitien d'Etudes du Sarcome de Kaposi et des Infections Opportunistes Port‐au‐Prince Haiti; ^8^ Department of Internal Medicine Faculty of Medicine University of Chile Santiago Chile; ^9^ Hospital Clínico San Borja Arriarán & Fundación Arriarán Santiago Chile; ^10^ Millenium Institute on Immunology and Immunotherapy Santiago Chile

**Keywords:** Caribbean Region, CD4 lymphocyte count, immune reconstitution, Latin America, morbidity, non‐communicable diseases

## Abstract

**Introduction:**

Immune reconstitution following antiretroviral therapy (ART) initiation is crucial to prevent AIDS and non‐AIDS‐related comorbidities. Patients with suppressed viraemia who fail to restore cellular immunity are exposed to an increased risk of morbidity and mortality during long‐term follow‐up, although the underlying mechanisms remain poorly understood. We aim to describe clinical outcomes and factors associated with the worse immune recovery and all‐cause mortality in people living with HIV (PLWH) from Latin America following ART initiation.

**Methods:**

Retrospective cohort study using the CCASAnet database: PLWH ≥18 years of age at ART initiation using a three drug‐based combination therapy and with medical follow‐up for ≥24 months after ART initiation and undetectable viral load were included. Patients were divided into four immune recovery groups based on rounded quartiles of increase in CD4 T‐cell count at 2 years of treatment (<150, [150, 250), [250, 350] and >350 cells/mm^3^). Primary outcomes included all‐cause mortality, AIDS‐defining events and non‐communicable diseases that occurred >2 years after ART initiation. Factors associated with an increase in CD4 T‐cell count at 2 years of treatment were evaluated using a cumulative probability model with a logit link.

**Results:**

In our cohort of 4496 Latin American PLWH, we found that patients with the lowest CD4 increase (<150) had the lowest survival probability at 10 years of follow‐up. Lower increase in CD4 count following therapy initiation (and remarkably not a lower baseline CD4 T‐cell count) and older age were risk factors for all‐cause mortality. We also found that older age, male sex and higher baseline CD4 T‐cell count were associated with lower CD4 count increase following therapy initiation.

**Conclusions:**

Our study shows that PLWH with lower increases in CD4 count have lower survival probabilities. CD4 increase during follow‐up might be a better predictor of mortality in undetectable PLWH than baseline CD4 count. Therefore, it should be included as a routine clinical variable to assess immune recovery and overall survival.

## 1 INTRODUCTION

According to the 2022 UNAIDS report, 38.4 million people worldwide were living with HIV at the end of 2021. Of those, 1.5 million people acquired HIV, and 650,000 people died of AIDS‐related complications. From a regional perspective, 2.2 million people living with HIV (PLWH) live in Latin America, 110,000 acquired the virus and 29,000 people died of AIDS‐related diseases during 2021 [1]. The global efforts to slow the HIV pandemic by widening access to antiretroviral therapy (ART) have been successful, but still, nearly 25% of all PLWH have no access to ART [1]. Furthermore, it has been reported that almost half of PLWH from Latin America start ART with a CD4 T‐cell count of <350 cells/ml [2], showing that late diagnosis persists as a problem. Both aforementioned factors are crucial to ensure viral undetectability and immune restoration once therapy is started [3, 4].

Immune performance following therapy initiation varies among patients. Specifically, some patients fail to restore CD4 T‐cell counts despite achieving viral undetectability [5, 6, 7]. Definitions for poor immune response are diverse and consider CD4 T‐cell count, CD4 T‐cell percentage, CD4/CD8 ratio and CD4 T‐cell count increase following ART initiation. As these parameters of immune response have different intra‐ and interindividual variations [8], a combined long‐term longitudinal assessment is required to appropriately classify patients. Regardless of the definition for poor immune response, this group of patients is exposed to an increased risk for all‐cause mortality [9, 10]. Therefore, the identification of this group is crucial. Some risk factors for poor immune response include older age at HIV diagnosis, male sex, late ART initiation and lower CD4 T‐cell nadir [8, 11, 12, 13]. Thus, early diagnosis and prompt therapy initiation are cornerstones to promote viral suppression and robust immune reconstitution [14, 15, 16].

In this retrospective cohort study, we aim to assess clinical outcomes and describe factors associated with the worse immune recovery and all‐cause mortality after ART initiation in Latin American PLWH.

## 2 METHODS

### 2.1 Study population

We conducted a retrospective cohort study using the Caribbean, Central and South American Network for HIV Epiodemiology (CCASAnet) database [17]. Participating CCASAnet sites were Instituto Nacional de Infectologia Evandro Chagas, Rio de Janeiro, Brazil (BR‐INI); Fundación Arriarán, Santiago, Chile (CL‐FA); Hospital Escuela and Instituto Hondureño de Seguridad Social, Tegucigalpa, Honduras (HN‐SS/HE); Instituto Nacional de Ciencias Médicas y Nutrición Salvador Zubirán, Mexico City, Mexico (MX‐INCMNSZ); and Instituto de Medicina Tropical Alexander von Humboldt, Universidad Peruana Cayetano Heredia, Lima, Peru (PE‐IMTAVH). The study included adults (≥18 years at ART initiation) with confirmed HIV diagnosis who started a three‐drug‐based combination ART regimen. Patients must have been under medical follow‐up at a CCASAnet HIV clinic for at least 2 years after ART initiation, received ART for at least 6 months without modification and had a sustained undetectable viral load (VL) between months 6−24 after ART initiation (i.e. all measurements <200 copies/ml). Patients with a history of one or two drug‐based ART regimens, undetectable viral load at ART initiation, no CD4 laboratory data at ART initiation or no CD4 laboratory data 2 years after ART initiation were excluded. Data were included on patients starting ART between October 1998 and January 2018. The ethical approval and the informed consent waiver for the use of these data were granted at each of the CCASAnet sites, and at Vanderbilt University Medical Center (IRB #060284).

### 2.2 Definitions of viro‐immunological performance, laboratory measurements and outcomes

We used the increase from baseline (ART initiation) in CD4 T‐cell count at 2 years of ART to characterize immunological performance. Patients were divided into four immune recovery groups based on rounded quartiles of increase in CD4 T‐cell count at 2 years of treatment (<150, [150, 250), [250, 350] and >350 cells/mm^3^). Baseline CD4 T‐cell count was defined as the measurement closest to the date of ART initiation within 6 months before or 1 month after. CD4 T‐cell count at 2 years was defined as the count on the day closest to 2 years after ART initiation within +/−3 months. Baseline viral load was defined as the measurement taken closest to ART initiation within 6 months before and 1 week after ART initiation. Primary outcomes included all‐cause mortality, AIDS‐defining events (opportunistic infections and AIDS‐defining cancers) and non‐AIDS‐defining events (cardiovascular diseases, such as cardiac and cerebrovascular events, and non‐AIDS‐defining cancers) that occurred >2 years after ART initiation.

### 2.3 Statistical analyses

Baseline characteristics, including demographic and HIV‐related information, were described for the four CD4‐change‐defined groups, individually and together. Differences in baseline characteristics between the four groups were tested with Kruskal−Wallis rank‐sum tests for continuous variables and Chi‐square tests for categorical variables.

Factors associated with an increase in CD4 T‐cell count at 2 years of treatment were evaluated using a cumulative probability model, which is a form of an ordinal logistic regression model [18]. CD4 increase was included as a continuous response variable in the cumulative probability model [18]. Explanatory variables were demographic and HIV‐related variables measured at ART initiation, including age, sex, HIV transmission, HIV centre, baseline CD4 T‐cell count, baseline viral load, history of tuberculosis, history of AIDS‐defining events, time from HIV diagnosis to ART initiation, type of starting regimens and whether starting regimens included zidovudine (AZT). Missing data were accounted for using multiple imputation with 10 imputation replications. To avoid linearity assumptions, we modelled continuous variables using restricted cubic splines (three knots for each variable).

We used Kaplan−Meier estimators to estimate survival probabilities for the four CD4‐change‐defined groups after ART initiation. The association between increase in CD4 T‐cell count after 2 years of treatment and all‐cause mortality was evaluated using a Cox proportional hazards model that stratified for HIV centre (i.e. allowing for different reference hazards per centre), adjusting for baseline CD4 T‐cell count and factors associated with CD4 change identified in the previous analysis. Baseline CD4 T‐cell count and increase in CD4 T‐cell count were modelled using restricted cubic splines with three knots. The model additionally included the linear interaction term of baseline CD4 T‐cell count and increase in CD4 T‐cell count. Furthermore, the cumulative incidences of AIDS‐defining events and non‐AIDS‐defining events for the four groups were estimated treating death as a competing event; differences in the risk of diseases between the four groups were compared using Gray's test [19]. In these analyses, we excluded patients who had specific diseases during the first 2 years of ART treatment. All analyses were performed with R statistical software version 4.0.3. The analysis code is available at https://biostat.app.vumc.org/ArchivedAnalyses .

## 3 RESULTS

### 3.1 Baseline characteristics

The CCASAnet database included 26,966 PLWH, of which 4496 met inclusion criteria. The consort diagram is reported in the Supplementary Material (Figure S1). The 4496 eligible PLWH were from sites in Brazil (30%), Chile (15%), Honduras (5%), Mexico (19%) and Peru (32%). Eligible participants were 80% male and had a median age of 35 years at ART initiation (interquartile range IQR 29−43). A total of 23% (*n* = 1048) had an increase in CD4 T‐cell count at 2 years of treatment of <150 cells/mm^3^, 25% (1102) had an increase from 150 to 249 cells/mm^3^, 21% (962) had an increase from 250 to 350 cells/mm^3^ and 31% (1384) had an increase >350 cells/mm^3^ (Table 1 and Figure S2). The four groups of PLWH had significant differences in some demographic and HIV‐related baseline characteristics, with men and older individuals tending to have lower CD4 increase.

**Table 1:** Baseline characteristics.

	Increase<150 (*N*=1048)	Increase=[150, 250) (*N*=1102)	Increase=[250, 350] (*N*=962)	Increase>350 (*N*=1384)	*p*‐value	Total (*N*=4496)
**Sex**						
Female	184 (17.6%)	207 (18.8%)	178 (18.5%)	331 (23.9%)	<0.001	900 (20.0%)
Male	864 (82.4%)	895 (81.2%)	784 (81.5%)	1053 (76.1%)		3596 (80.0%)
**Age at ART initiation (years)**						
Mean (SD)	38.1 (11.2)	36.9 (10)	36 (10)	35.1 (9.6)	<0.001	36.5 (10.3)
Median [Q1, Q3]	36.8 [29.7, 45]	35.4 [29.4, 43.2]	34.6 [28.7, 41.8]	33.8 [27.8, 41.4]		34.8 [28.7, 42.8]
**HIV centre**						
BR‐IPEC	251 (24.0%)	245 (22.2%)	282 (29.3%)	585 (42.3%)	<0.001	1363 (30.3%)
CL‐FA	186 (17.7%)	198 (18.0%)	118 (12.3%)	153 (11.1%)		655 (14.6%)
HN‐SS/HE	81 (7.7%)	55 (5.0%)	39 (4.1%)	31 (2.2%)		206 (4.6%)
MX‐INCMNSZ	210 (20.0%)	259 (23.5%)	186 (19.3%)	188 (13.6%)		843 (18.8%)
PE‐IMTAVH	320 (30.5%)	345 (31.3%)	337 (35.0%)	427 (30.9%)		1429 (31.8%)
**HIV transmission route**						
Heterosexual contact	416 (39.7%)	433 (39.3%)	374 (38.9%)	576 (41.6%)	0.564	1799 (40.0%)
Homosexual/bisexual contact	563 (53.7%)	615 (55.8%)	540 (56.1%)	734 (53.0%)		2452 (54.5%)
Other	16 (1.5%)	14 (1.3%)	10 (1.0%)	13 (0.9%)		53 (1.2%)
Unknown	53 (5.1%)	40 (3.6%)	38 (4.0%)	61 (4.4%)		192 (4.3%)
**History of tuberculosis**						
No	928 (88.5%)	969 (87.9%)	834 (86.7%)	1197 (86.5%)	0.39	3928 (87.4%)
Yes	120 (11.5%)	133 (12.1%)	128 (13.3%)	187 (13.5%)		568 (12.6%)
**AIDS‐defining events prior to ART initiation**						
No	638 (60.9%)	580 (52.6%)	525 (54.6%)	781 (56.4%)	<0.001	2524 (56.1%)
Yes	311 (29.7%)	428 (38.8%)	359 (37.3%)	497 (35.9%)		1595 (35.5%)
Unknown	99 (9.4%)	94 (8.5%)	78 (8.1%)	106 (7.7%)		377 (8.4%)
**Viral load at ART initiation (10^5^ copies/ml)**						
Mean (SD)	2.5 (9.4)	2.3 (6.1)	3.2 (7.4)	3.7 (10.3)	<0.001	3 (8.6)
Median [Q1, Q3]	0.7 [0.1, 1.6]	0.8 [0.3, 2.1]	1 [0.3, 2.9]	1 [0.3, 3.3]		0.8 [0.2, 2.4]
Missing	180 (17.2%)	150 (13.6%)	161 (16.7%)	184 (13.3%)	0.01^a^	675 (15.0%)
**CD4 T‐cell count at ART initiation (cells/mm^3^)**						
Mean (SD)	288 (236.4)	196 (165.2)	200 (167.5)	227 (181.2)	<0.001	228 (192.5)
Median [Q1, Q3]	244 [120, 370]	168 [55, 294]	175 [58, 296]	207 [78, 324]		199 [74, 321]
**Days from HIV diagnosis to ART initiation**						
Mean (SD)	653.9 (1101.1)	546.6 (985.4)	563.2 (896.3)	596.4 (1016.1)	0.088	590.6 (1005.6)
Median [Q1, Q3]	142 [56, 753]	125 [47.2, 543.7]	144 [50, 683]	123 [47, 721]		134 [50, 685.2]
Missing	3 (0.3%)	8 (0.7%)	2 (0.2%)	3 (0.2%)		16 (0.4%)
**Type of starting regimens**						
Protease inhibitors	93 (8.9%)	130 (11.8%)	132 (13.7%)	216 (15.6%)	<0.001	571 (12.7%)
Non‐nucleoside reverse transcriptase inhibitors	926 (88.4%)	951 (86.3%)	805 (83.7%)	1130 (81.6%)		3812 (84.8%)
Integrase inhibitors	29 (2.8%)	21 (1.9%)	24 (2.5%)	38 (2.7%)		112 (2.5%)
Missing	0 (0%)	0 (0%)	1 (0.1%)	0 (0%)		1 (0.0%)
**Starting regimens included AZT**						
Yes	561 (53.5%)	554 (50.3%)	515 (53.5%)	674 (48.7%)	0.042	2304 (51.2%)
No	487 (46.5%)	548 (49.7%)	447 (46.5%)	710 (51.3%)		2192 (48.8%)
**Follow‐up years**						
Mean (SD)	7.1 (3.6)	7.6 (3.5)	7.4 (3.6)	7.2 (3.3)	0.002	7.3 (3.5)
Median [Q1, Q3]	6.2 [4.3, 9.3]	7 [4.7, 9.9]	6.8 [4.4, 9.8]	6.7 [4.4, 9.3]		6.7 [4.5, 9.6]
**Calendar year of HIV diagnosis** ^b^						
Mean (SD)	2009 (5.2)	2009 (4.9)	2009 (4.6)	2010 (4.6)	<0.001	2009 (4.8)
Median [Q1, Q3]	2010 [2006, 2013]	2010 [2006, 2012]	2010 [2006, 2013]	2010 [2007, 2013]		2010 [2006, 2013]
Missing	3 (0.3%)	8 (0.7%)	2 (0.2%)	3 (0.2%)		16 (0.4%)
**Calendar year of ART initiation**						
Mean (SD)	2011 (4)	2010 (3.9)	2011 (3.8)	2011 (3.5)	<0.001	2011 (3.8)
Median [Q1, Q3]	2011 [2008, 2013]	2011 [2008, 2013]	2011 [2008, 2014]	2012 [2009, 2014]		2011 [2009, 2014]
**Total number of viral load measurements during the follow‐up**						
Mean (SD)	13.5 (8.5)	14.8 (8.7)	14.6 (8.9)	14.3 (8.6)	0.001	14.3 (8.7)
Median [Q1, Q3]	12 [7, 18]	13 [8, 19]	13 [8, 19]	12 [8, 19]		12 [8, 19]
**Number of viral load measurements from months 6 to 24**						
Mean (SD)	3 (1.2)	3.2 (1.4)	3.2 (1.3)	3.3 (1.3)	<0.001	3.2 (1.3)
Median [Q1, Q3]	3 [2, 4]	3 [2, 4]	3 [2, 4]	3 [3, 4]		3 [2, 4]

*Note*: The less frequent probable routes of HIV acquisition were collapsed, including injecting drug user, homo/bisexual and injecting drug user, transfusion non‐haemophilia related, perinatal and other.

Abbreviations: ART, antiretroviral therapy; AZT, zidovudine; BR‐INI, Brazil CCASAnet site; CL‐FA, Chile CCASAnet site; HN‐SS/HE, Honduras CCASAnet site; MX‐INCMNSZ, Mexico CCASAnet site; PE‐IMTAVH, Peru CCASAnet site; Q1, first quartile; Q3, third quartile; SD, standard deviation.

^a^ Unadjusted test of the difference in baseline viral load missingness between the four groups.

^b^ A histogram showing the distribution of year of HIV diagnosis is shown in Figure S2.

### 3.2 Factors associated with worse immunological performance

**Figure 1:**
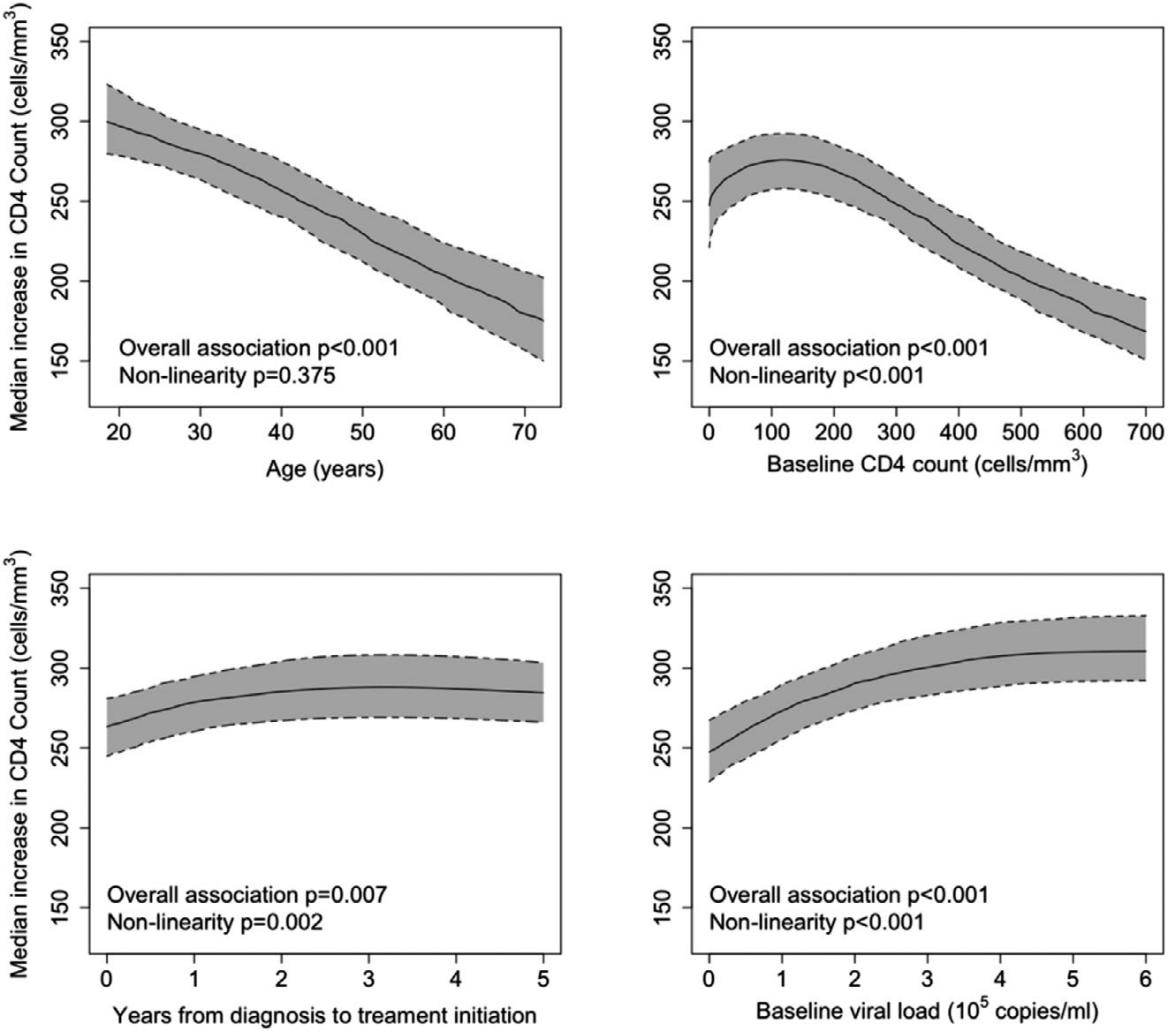
The association between continuous factors and median increase in CD4 T‐cell count at 2 years; all other variables are kept at their median or mode levels.

The association between patient factors and an increase in CD4 T‐cell count at 2 years of treatment is given in Table 2. Younger age at ART initiation, lower baseline CD4 T‐cell count, longer time from diagnosis to ART initiation, higher baseline viral load, female sex, starting regimens without AZT and starting a protease inhibitor (PI)‐based regimen (compared to a Non‐Nucleoside Reverse Transcriptase Inhibitor (NNRTI)‐based regimen) were associated with greater CD4 T‐cell count increase after 2 years of treatment. Furthermore, baseline CD4 T‐cell count, time from diagnosis to ART initiation and baseline viral load had significant non‐linear relationships with an increase in CD4 T‐cell count (Figure 1), adjusting for other covariates.

**Table 2:** Adjusted odds ratios for greater CD4 T‐cell increase at 2 years.

Predictors	Odds ratio	95% CI	*p*‐value
Age (years)			<0.001
20 versus 35	1.34	[1.12, 1.61]	
30 versus 35	1.11	[1.06, 1.16]	
40 versus 35	0.89	[0.86, 0.91]	
50 versus 35	0.67	[0.61, 0.74]	
Baseline CD4 count (cells/mm^3^)			<0.001
50 versus 200	1.00	[0.91, 1.11]	
100 versus 200	1.06	[1.01, 1.11]	
300 versus 200	0.81	[0.78, 0.85]	
500 versus 200	0.50	[0.43, 0.58]	
Time from diagnosis to ART initiation			0.007
0 versus 6 months	0.92	[0.87, 0.97]	
3 versus 6 months	0.96	[0.94, 0.98]	
1 year versus 6 months	1.07	[1.03, 1.12]	
2 years versus 6 months	1.16	[1.06, 1.27]	
Baseline viral load (10^5^ copies/ml)			<0.001
0.01 versus 0.6	0.86	[0.82, 0.90]	
0.1 versus 0.6	0.88	[0.85, 0.91]	
3 versus 0.6	1.47	[1.32, 1.64]	
5 versus 0.6	1.60	[1.41, 1.83]	
Female versus male	1.40	[1.20, 1.63]	<0.001
Heterosexual contact versus homo/bisexual/other contact	0.93	[0.81, 1.06]	0.279
TB history: yes versus no	0.88	[0.73, 1.05]	0.143
AIDS‐defining events: yes versus no	1.05	[0.92, 1.21]	0.451
Starting regimens included AZT: yes versus no	0.88	[0.79, 0.99]	0.037
Starting regimen			<0.001
PI versus NNRTI	1.42	[1.21, 1.66]	
II versus NNRTI	1.00	[0.71, 1.41]	
HIV centre			<0.001
BR‐IPEC versus PE‐IMTAVH	1.75	[1.51, 2.02]	
CL‐FA versus PE‐IMTAVH	0.67	[0.56, 0.80]	
HN‐HE/SS versus PE‐IMTAVH	0.47	[0.36, 0.61]	
MX‐INCMNSZ versus PE‐IMTAVH	0.71	[0.60, 0.84]	

*Note*: The CD4 count increase was included as a continuous outcome in the cumulative probability model. Because it was modelled ordinally, associations with predictors are presented using odds ratios. The odds ratios compare two groups with respect to the odds of having a greater increase in CD4 T‐cell count at 2 years of treatment.

Abbreviations: AZT, zidovudine; BR‐INI, Brazil CCASAnet site; CL‐FA, Chile CCASAnet site; HN‐SS/HE, Honduras CCASAnet site; MX‐INCMNSZ, Mexico CCASAnet site; PE‐IMTAVH, Peru CCASAnet site; TB, tuberculosis.

### 3.3 Assessment of all‐cause mortality during follow‐up

**Figure 2:**
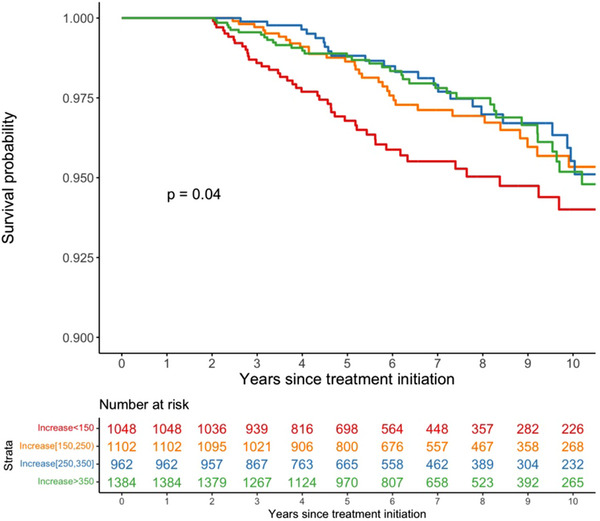
Survival probability based on CD4‐cell count increases at the first 2 years of ART initiation over 10 years after ART initiation. The *p*‐value is from a log‐rank test for the difference in survival probability between the four groups.

**Figure 3:**
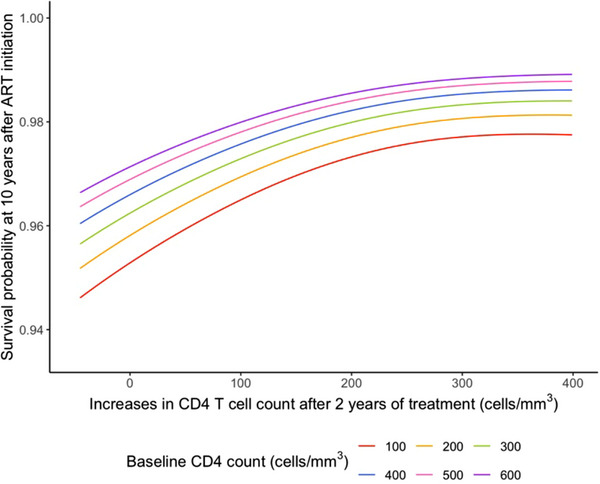
The association between increase in CD4 T‐cell count at 2‐year treatment and baseline CD4 T‐cell count with survival probability at 10 years after ART treatment initiation; other variables are set to their median or mode levels.

There were 146 (3.2%) patients who died during follow‐up. Patients with an increase of <150 cells/mm^3^ at 2 years had the lowest survival probability during the 10 years after ART initiation (i.e. 8 years after eligibility for our study) (Figure 2). A lower increase in CD4 T‐cell count over 2 years was associated with a higher risk of all‐cause mortality, adjusting for baseline CD4 T‐cell count, age, time from diagnosis to ART initiation, sex, starting regimens and HIV centre (Table 3 and Figure 3). The association between increase in baseline CD4 T‐cell count and all‐cause mortality was significantly non‐linear (*p* = 0.001, Table S1), but the interaction between baseline CD4 T‐cell count and increase in CD4 T‐cell count was not significant (*p* = 0.30, Table S1).

**Table 3:** Adjusted hazard ratios of the Cox proportional hazard model for all‐cause mortality.

Predictors	Hazard ratio	95% CI	*p*‐value
Increase in CD4 count (cells/mm^3^)			<0.001
0 versus 200	1.84	[1.33, 2.54]	
100 versus 200	1.34	[1.15, 1.56]	
300 versus 200	0.84	[0.76, 0.93]	
500 versus 200	0.85	[0.66, 1.11]	
Baseline CD4 count (cells/mm^3^)			0.064
50 versus 200	1.31	[1.00, 1.72]	
100 versus 200	1.18	[1.03, 1.35]	
300 versus 200	0.87	[0.74, 1.01]	
500 versus 200	0.68	[0.40, 1.14]	
Age (years)			<0.001
20 versus 35	0.40	[0.32, 0.50]	
30 versus 35	0.74	[0.69, 0.79]	
40 versus 35	1.35	[1.26, 1.46]	
50 versus 35	2.48	[1.99, 3.09]	
Time from diagnosis to ART initiation			0.301
0 versus 6 months	0.99	[0.96, 1.01]	
3 versus 6 months	0.99	[0.98, 1.01]	
1 year versus 6 months	1.01	[0.99, 1.04]	
2 years versus 6 months	1.04	[0.96, 1.12]	
Baseline viral load (10^5^ copies/ml)			0.660
0.01 versus 0.6	1.00	[0.99, 1.02]	
0.1 versus 0.6	1.00	[0.99, 1.02]	
3 versus 0.6	0.98	[0.92, 1.06]	
5 versus 0.6	0.97	[0.85, 1.11]	
Female versus male	0.67	[0.43, 1.04]	0.072
Starting regimens included AZT: yes versus no	1.14	[0.79, 1.65]	0.484
Starting regimens			0.063
PI versus NNRTI	1.66	[1.09, 2.53]	
II versus NNRTI	0.99	[0.14, 7.27]	

There was one (1%) death among 112 patients starting an integrase inhibitor (II)‐based regimen, compared to 114 (3%) deaths among 3812 patients starting an Non‐Nucleoside Reverse Transcriptase Inhibitor (NNRTI)‐based regimen and 31 (5%) deaths among 571 patients starting a PI‐based regimen. The reported associations between baseline CD4 and mortality hold change in CD4 constant at its median, 259 cells/mm^3^, and the associations between change in CD4 and mortality hold baseline CD4 constant at its median, 199 cells/mm^3^.

### 3.4 Risks of specific diseases

**Figure 4:**
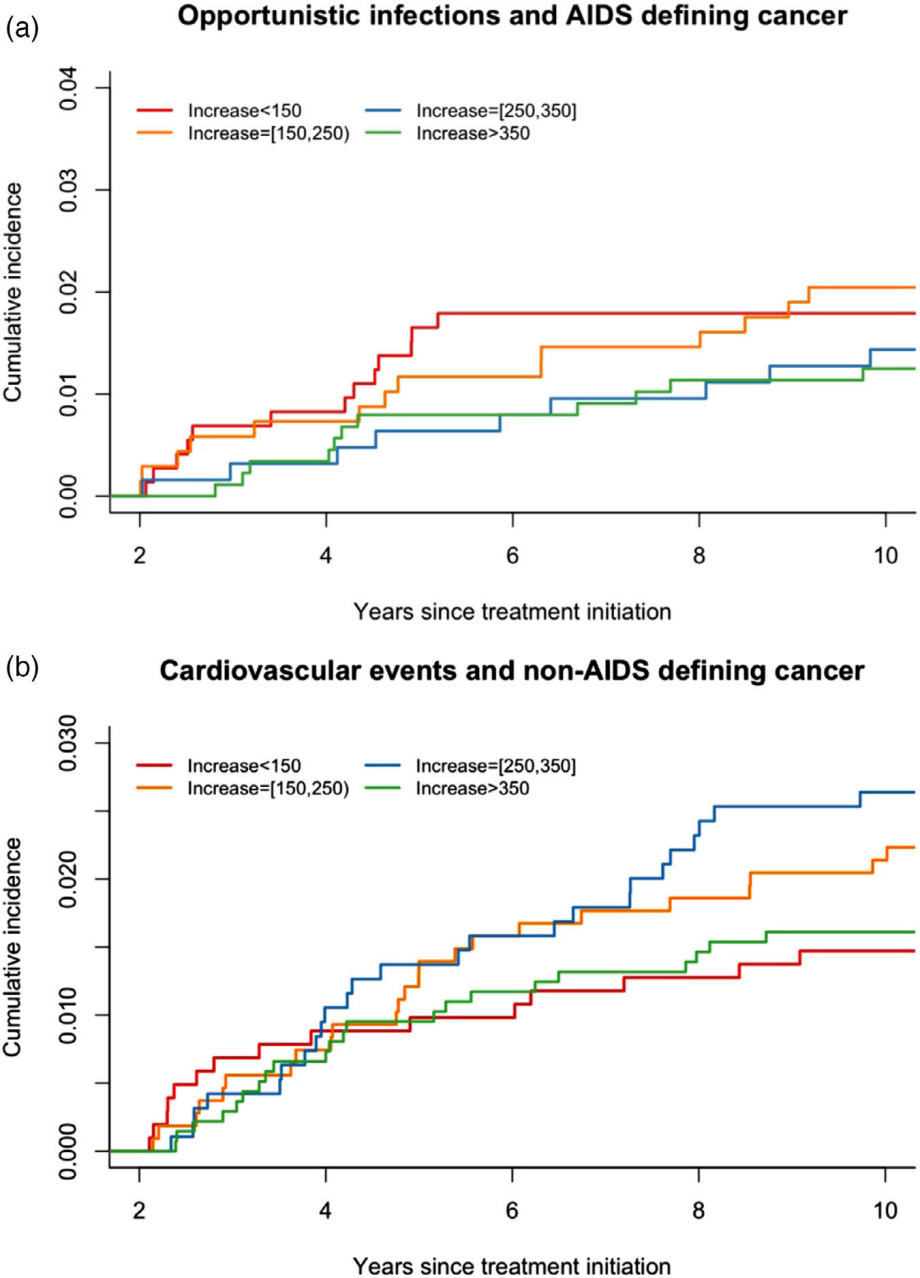
(a) Cumulative incidence of opportunistic infections and AIDS‐defining cancer in 10 years of ART. (b) Cumulative incidence of cardiovascular events and non‐AIDS‐defining cancer in 10 years of ART.

A total of 2917 patients were eligible in the analysis estimating the risk of AIDS‐defining events (i.e. did not have an AIDS‐defining event during the first 2 years of ART). Of these patients, 50 (1.7%) developed AIDS ≥2 years after ART initiation, and 57 (2.0%) died without having AIDS (Table 4). The difference in the risk between the four groups was not significant (*p* = 0.51, Figure 4A and Table S2). In the analysis for cardiovascular events and non‐AIDS‐defining cancers, a total of 4407 patients were eligible (i.e. not having a cardiovascular or non‐AIDS‐defining cancer event during the first 2 years of ART). Of these patients, 93 (2.1%) developed an event ≥2 years after ART initiation, and 113 (2.6%) patients died before having a cardiovascular or non‐AIDS cancer event. We did not find sufficient statistical evidence to conclude that there was a difference in the cumulative incidence of cardiovascular events and non‐AIDS‐defining cancers among the four groups (*p* = 0.45, Figure 4B and Table S2).

**Table 4:** Number of patients with specific diseases in each group.

	Increase<150	Increase=[150, 250)	Increase=[250, 350]	Increase>350	Total
**Opportunistic infections and AIDS‐defining cancer**					
Censor	688 (94.77%)	657 (96.05%)	610 (97.29%)	855 (97.16%)	2810 (96.33%)
Event	14 (1.93%)	15 (2.19%)	10 (1.59%)	11 (1.25%)	50 (1.71%)
Death	24 (3.31%)	12 (1.75%)	7 (1.12%)	14 (1.59%)	57 (1.95%)
Total	726	684	627	880	2917
**Cardiovascular events and non‐AIDS‐defining cancer**					
Censor	966 (94.8%)	1020 (94.88%)	904 (95.36%)	1311 (96.04%)	4201 (95.33%)
Event	19 (1.86%)	25 (2.33%)	25 (2.64%)	24 (1.76%)	93 (2.11%)
Death	34 (3.34%)	30 (2.79%)	19 (2%)	30 (2.2%)	113 (2.56%)
Total	1019	1075	948	1365	4407

## 4 DISCUSSION

For the first time in a large Latin American cohort of treated PLWH with virologic success, we describe clinical outcomes and risk factors for worse immune recovery and for all‐cause mortality. We found that patients with an increase of <150 cells/ml 2 years after ART initiation had the lowest survival probability among those with a sustained undetectable VL. In a multivariable model, a lower increase in CD4 count, older age and starting treatment with a PI‐including regimen were associated with a higher risk of death after 2 years. The latter results might be explained by the use of PIs in patients with more advanced disease at diagnosis in the era prior to integrase inhibitors rollout in Latin America. Of note, CD4 T‐cell count at baseline (prior to ART initiation) was not strongly associated with increased mortality after adjusting for CD4 change over the first 2 years of ART. This result highlights the relevance of the increase in CD4 T‐cell count for predicting mortality, in contrast to the *nadir* of CD4 at baseline. Therefore, CD4 T‐cell count change should be included as a relevant clinical parameter to be considered during the follow‐up for patients with virological success.

In terms of immune performance, younger age at ART initiation, female sex, lower CD4 count at baseline and PI‐including regimen at treatment initiation were associated with a higher increase in CD4 count following 2 years of treatment with undetectable viral load. Interestingly, we also found that a shorter time from diagnosis to ART initiation and a lower baseline viral load were also associated with a lower increase in CD4 count. Although unexpected, these results might be due to a worse clinical status at HIV diagnosis in our cohort (opportunistic infections and AIDS‐related diseases), especially considering that prior to 2016, treatment initiation was decided according to progression to AIDS instead of a “test and treat” strategy that is currently recommended. In fact, 93.6% of the patients from the cohort were diagnosed prior to the “Treat‐All Era” (Figure S3). This fact also helps to explain the low rate of integrase strand transfer inhibitors (INSTI) use in our cohort. On the other hand, we found different probabilities for immune recovery among the participating sites. This result might be partially explained by differences in CD4 T‐cell count and viral load monitoring [20], as well as by other uncontrolled variables, such as viral coinfections [21].

Noteworthy, the survival probability of the entire cohort proved to be high (over 90% at 10 years) despite the differences in the increase of CD4 count. This might suggest that uncontrolled or untreated HIV infection plays the most important role as a predictor for mortality, with CD4 count having less influence, once viral undetectability has been reached and a patient successfully makes it through the first 2 years of ART.

Nevertheless, some limitations must be acknowledged. This cohort is composed of patients proceeding from five different countries and HIV centres, with different access to ART regimens and with different levels of database completion. Therefore, some desirable exclusion criteria could not be used, such as hepatitis B and C virus co‐infection, as not all HIV centres tested routinely for these viruses. In the same way, the different relative contribution from each centre could have influenced results by overrepresenting some clinical effects. Furthermore, information bias due to lack of database completion could have diverted some of our results, but imputation techniques for missing data were used. It must be considered that our parameter to evaluate immune performance was the increase in CD4 T‐cell count at 2 years of ART initiation. However, there are other parameters that can help to better characterize immune performance, such as the CD4/CD8 ratio. Unfortunately, this parameter was not available, as most HIV centres in our cohort do not routinely register CD8 count. The CD4/CD8 ratio is recognized as a good marker for immune recovery during follow‐up and it could be used as a predictor for morbidity and mortality in undetectable patients [10, 12, 22, 23]. Therefore, routine registration of CD8 count and CD4/CD8 could help to assess immune recovery and to predict non‐AIDS complications and mortality in the long‐term. Likewise, its inclusion as a routine parameter for clinical evaluation might help to optimize immune recovery assessment for future research. Finally, most of our cohort was diagnosed prior to the “Treat‐All era.” In consequence, results in terms of morbidity, mortality and factors for immune recovery may have differed if we had only focused on patients diagnosed from 2015 to 2016 onwards. Further research on immune recovery in the “Treat‐All era” is warranted.

## 5 CONCLUSIONS

In summary, this retrospective cohort analysis of undetectable Latin American PLWH on ART allowed us to understand some risk factors for mortality and immune recovery in successfully treated patients in our region.

We highlight two messages: first, that the increase in CD4 count during follow‐up should be included as a clinical variable to assess immune recovery and, particularly, the risk for mortality, as it proved to be a more important predictor than baseline CD4 count. And second, that these results might work as a reinforcement for viral undetectability as the most important goal of treatment, to ensure better clinical long‐term outcomes.

## COMPETING INTERESTS

No competing interests were reported.

## AUTHORS’ CONTRIBUTIONS

GC‐R and CPC designed the research study. ST and BES performed the statistical analyses. GC‐R and ST wrote the paper. GC‐R, ST, FM, VR, PML, BES and CPC reviewed the manuscript critically and approved its final version.

## FUNDING

This work was supported by the NIH‐funded Caribbean, Central and South America network for HIV epidemiology (CCASAnet), a member cohort of the International Epidemiologic Databases to Evaluate AIDS (leDEA) (U01AI069923) and Fondo de Financiamiento de Centros de Investigación en Áreas Prioritarias ATE 220016, ANID, Chile.

## DISCLAIMER

The content is solely the responsibility of the authors and does not necessarily represent the official views of the National Institutes of Health.

## Supporting information


**Table S1**. Wald tests of predictors in the Cox proportional hazard model for all‐cause mortality.
**Table S2**. Number of patients with specific diseases in each country.
**Figure S1**. The diagram of applying inclusion and exclusion criteria.
**Figure S2**. Longitudinal CD4 T‐cell counts in 2 years after ART initiation. The black solid curves are natural cubic splines with 5 degrees of freedom.
**Figure S3**. Histogram showing the patients' year of HIV diagnosis. 93.6% of the cohort was diagnosed prior to the “Treat‐All” era.

## Data Availability

The data that support the findings of this study are available from the corresponding author upon reasonable request.
