# Supplementary material for: Clinical outcomes and risk factors for immune recovery and all‐cause mortality in Latin Americans living with HIV with virological success: a retrospective cohort study
Source: J Int AIDS Soc. 2024 Mar 17;27(3):e26214. doi: 10.1002/jia2.26214 (PMC10945036; doi:10.1002/jia2.26214)
Supplement: Supplementary file 1 — Table S1. Wald tests of predictors in the Cox proportional hazard model for all‐cause mortality. Table S2. Number of patients with specific diseases in each country. Figure S1. The diagram of applying inclusion and exclusion criteria. Figure S2. Longitudinal CD4 T‐cell counts in 2 years after ART initiation. The black solid curves are natural cubic splines with 5 degrees of freedom. Figure S3. Histogram showing the patients' year of HIV diagnosis. 93.6% of the cohort was diagnosed prior to the “Treat‐All” era. [file JIA2-27-e26214-s001.docx]

# Supplementary Material

**Table S1.** Wald tests of predictors in the Cox proportional hazard model for all-cause mortality

|  | Chi-Square | d.f. | P-value |
| --- | --- | --- | --- |
| Increase in CD4 count (Factor+Higher Order Factors) | 24.72 | 3.00 | <0.001 |
| Nonlinear | 10.13 | 1.00 | 0.001 |
| Baseline CD4 count (Factor+Higher Order Factors) | 7.26 | 3.00 | 0.064 |
| Nonlinear | 0.05 | 1.00 | 0.818 |
| Increase in CD4 count * Baseline CD4 count (Factor+Higher Order Factors) | 1.12 | 1.00 | 0.290 |
| Age | 66.06 | 1.00 | <0.001 |
| Time from diagnosis to ART initiation | 1.07 | 1.00 | 0.301 |
| Baseline viral load | 0.19 | 1.00 | 0.660 |
| Sex | 3.23 | 1.00 | 0.072 |
| Starting regimens included AZT | 0.49 | 1.00 | 0.484 |
| Type of starting regimens | 5.54 | 2.00 | 0.063 |
| TOTAL NONLINEAR | 10.57 | 2.00 | 0.005 |
| TOTAL NONLINEAR + INTERACTION | 18.46 | 3.00 | <0.001 |
| TOTAL | 117.20 | 12.00 | <0.001 |

**Table S2.** Number of patients with specific diseases in each country

|  | BRA | CHL | HND | MEX | PER | Total |
| --- | --- | --- | --- | --- | --- | --- |
| **Opportunistic infections and AIDS defining cancer** | | | | | | |
| Censor | 817 (95.67%) | 439 (95.85%) | 70 (86.42%) | 507 (96.39%) | 977 (97.9%) | 2810 (96.33%) |
| Event | 16 (1.87%) | 5 (1.09%) | 9 (11.11%) | 11 (2.09%) | 9 (0.9%) | 50 (1.71%) |
| Death | 21 (2.46%) | 14 (3.06%) | 2 (2.47%) | 8 (1.52%) | 12 (1.2%) | 57 (1.95%) |
| Total | 854 | 458 | 81 | 526 | 998 | 2917 |
| **Cardiovascular events and Non-AIDS defining cancer** | | | | | | |
| Censor | 1239 (93.37%) | 609 (94.27%) | 197 (96.1%) | 764 (94.44%) | 1392 (98.03%) | 4201 (95.33%) |
| Event | 44 (3.32%) | 11 (1.7%) | 1 (0.49%) | 33 (4.08%) | 4 (0.28%) | 93 (2.11%) |
| Death | 44 (3.32%) | 26 (4.02%) | 7 (3.41%) | 12 (1.48%) | 24 (1.69%) | 113 (2.56%) |
| Total | 1327 | 646 | 205 | 809 | 1420 | 4407 |

BRA: Brazil CCASAnet site, CHL: Chile CCASAnet site, HND: Honduras CCASAnet site, MEX: Mexico CCASAnet site, PER: Peru CCASAnet site.

# Figure S1. The diagram of applying inclusion and exclusion criteria

26,966 PLWH in CCASAnet Database

- 65 PLWH had no VL measurements
- 3,283 had detectable VL after 6 months in 2 years of ART
- 781 PLWH with age at the date of treatment initiation <18 years old
- 4,493 under medical follow-up for <18 months after ART initiation
- 67 received a 3 drug-based ART regime for <6 months without modification
- 618 PLWH had no CD4 lab between 180 days before and 30 days after treatment initiation.
- 1,963 had no CD4 lab between 24$\pm$3 months after treatment initiation.

- 3,998 PLWH never started ART
- 3,327 had a history of a 1 or 2 drug-based ART regimes
- 3,516 had a history of prior ART use
- 359 had an undetectable viral load at baseline (evidence they were on prior ART)

4,496 PLWH

7,077 PLWH

10,425 PLWH

15,766 PLWH


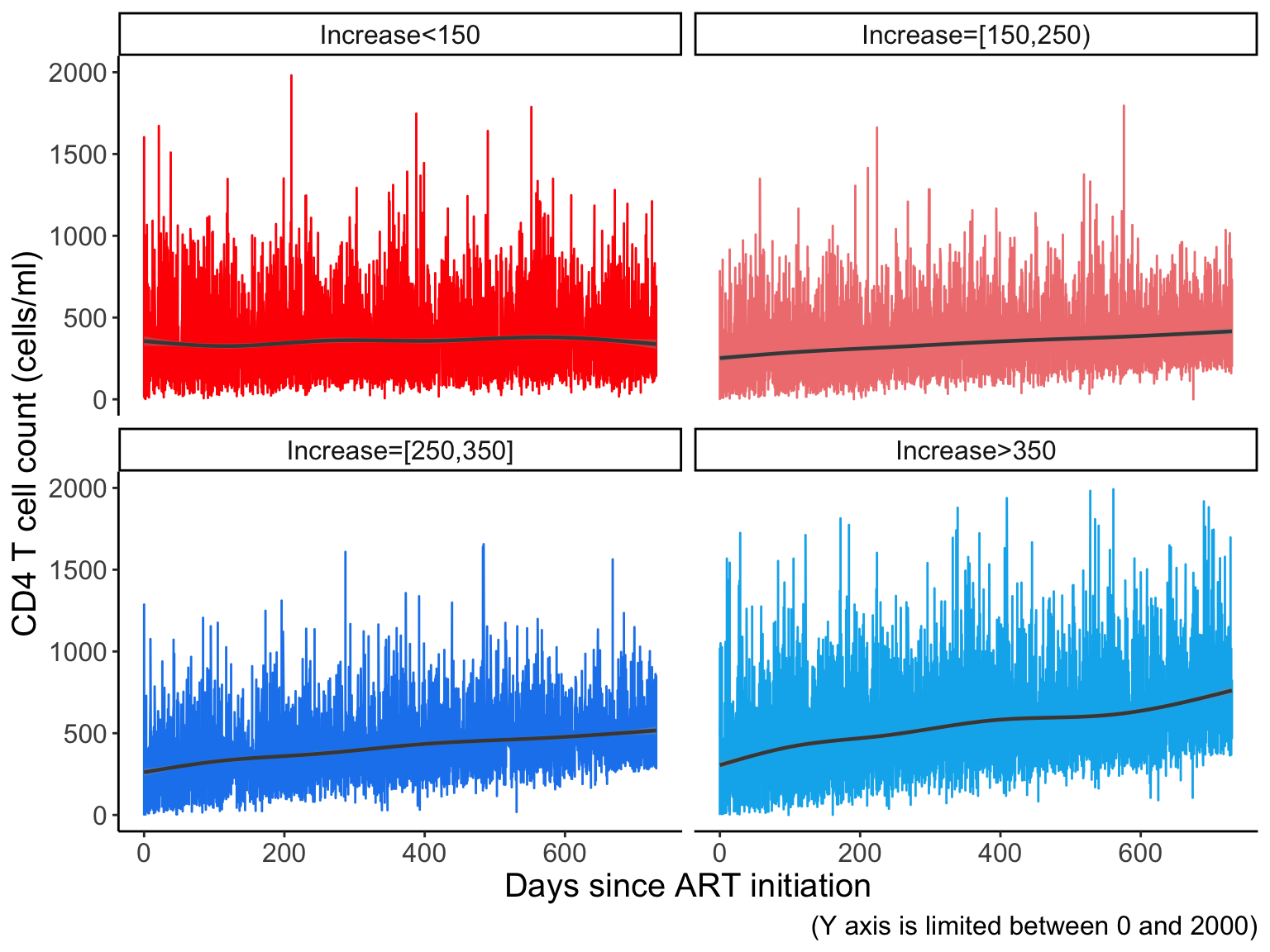


**Figure S2.** Longitudinal CD4 T-cell counts in 2 years after ART initiation.

The black solid curves are natural cubic splines with 5 degrees of freedom.

**
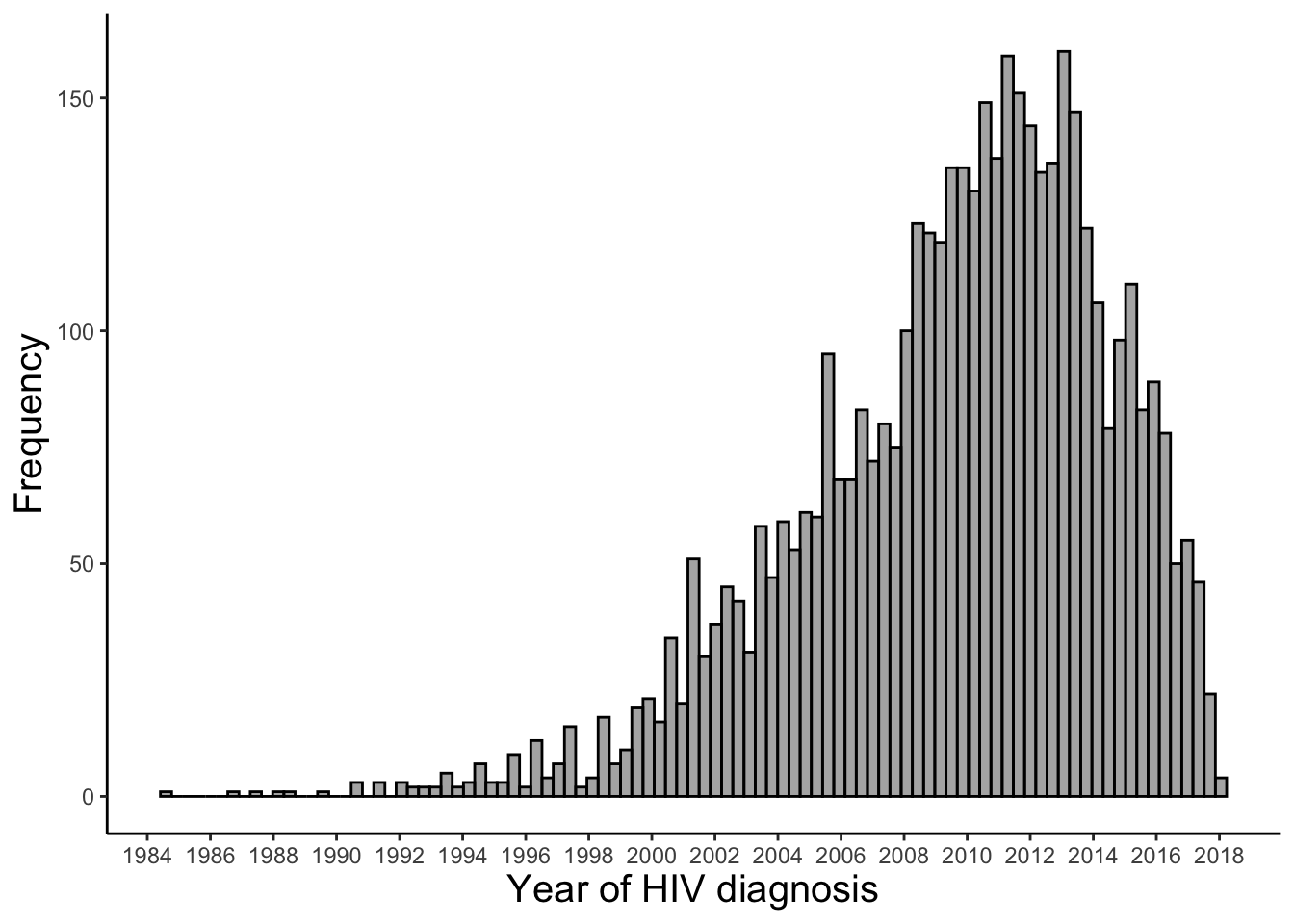
**

**Figure S3.** Histogram showing the patients’ year of HIV diagnosis. 93.6% of the cohort was diagnosed prior to the “Treat-All” era.
